# Paraquat Poisoning Induces TNF-α-Dependent iNOS/NO Mediated Hyporesponsiveness of the Aorta to Vasoconstrictors in Rats

**DOI:** 10.1371/journal.pone.0073562

**Published:** 2013-09-09

**Authors:** Rosária D. Aires, Luciano S. A. Capettini, Josiane F. Silva, Maria da Glória Rodrigues-Machado, Vanessa Pinho, Mauro M. Teixeira, Steyner F. Cortes, Virginia S. Lemos

**Affiliations:** 1 Department of Physiology and Biophysics, ICB, Federal University of Minas Gerais, Belo Horizonte, Minas Gerais, Brazil; 2 Department of Pharmacology, ICB, Federal University of Minas Gerais, Belo Horizonte, Minas Gerais, Brazil; 3 Department of Morphology, ICB, Federal University of Minas Gerais, Belo Horizonte, Minas Gerais, Brazil; 4 Department of Biochemistry and Immunology, ICB, Federal University of Minas Gerais, Belo Horizonte, Minas Gerais, Brazil; Kaohsiung Chang Gung Memorial Hospital, Taiwan

## Abstract

Paraquat is a toxic herbicide that may induce acute lung injury, circulatory failure and death. The present work aimed at investigating whether there is systemic inflammation and vascular dysfunction after paraquat exposure and whether these parameters were related. There was neutrophilia and accumulation of neutrophils in lung and bronchoalveolar lavage of animals given paraquat. This was associated with an increase in serum levels of TNF-α. In rats given paraquat, the relaxant response of aortic rings to acetylcholine was not modified but the contractile response to phenylephrine was greatly reduced. Endothelium removal or treatment with non-selective (L-NAME) or selective (L-NIL) inhibitors of inducible nitric oxide synthase (iNOS) restored contraction of aortas. There was greater production of nitric oxide (NO), which was restored to basal level by L-NIL, and greater expression of iNOS in endothelial cells, as seen by Western blot analyses and confocal microscopy. Blockade of TNF-α reduced pulmonary and systemic inflammation and vascular dysfunction. Together, our results clearly show that paraquat causes pulmonary and systemic inflammation, and vascular dysfunction in rats. Vascular dysfunction is TNF-α dependent, associated with enhanced expression of iNOS in aortic endothelial cells and greater NO production, which accounts for the decreased responsiveness of aortas to vasoconstrictors. Blockers of TNF-α may be useful in patients with paraquat poisoning.

## Introduction

Paraquat (1,1’-dimethyl-4,4’-bipyridinium dichloride) is a non-selective and contact herbicide used worldwide and cause high mortality rate (more than 50%) after accidental or deliberate self-poisoning [1]. Acute lung injury (ALI) is the main consequence of such poisoning due to active polyamine uptake transport systems that concentrate paraquat rapidly into type II epithelial cells of alveoli [2,3,4]. The mechanism of paraquat-induced cytotoxicity is not completely clear, but it is known that paraquat undergoes a redox cycling reaction, resulting in the oxidation of NADPH to NADP^+^, which lead to the production of reactive oxygen species [5] causing lipid peroxidation [6], cell damage [7] and consequent inflammatory reaction [8].

Acute lung injury is known to cause changes in the pulmonary vasculature. The balance between vasodilators and vasoconstrictors is disrupted, resulting in a disturbance in vascular resistance [9]. Vascular dysfunction associates with an intense neutrophilic response in the lungs seen after various stimuli and cytokines, such as TNF-α, appear to play a major pathogenic role [10,11]. The mechanism underling vascular malfunctioning in paraquat intoxication is largely unknown and most studies have focused on direct *in vitro* vascular effects of paraquat. In this regard, it is well known that paraquat is capable of producing superoxide, hence decreasing endothelium-dependent vasorelaxant responses [12,13]. However, these data are not in line with the vascular collapse that follows paraquat intoxication and that greatly contributes to early mortality of patients with paraquat poisoning [14,15]. To date there are no studies focusing on systemic inflammatory response and the impact for the systemic vascular responsiveness after toxic exposure to paraquat. The purpose of this study was to evaluate systemic inflammatory response and systemic vascular responsiveness during paraquat intoxication. A better understanding of the mechanisms involved in vascular alterations induced by paraquat poisoning may lead to more effective therapies.

## Materials and Methods

### Ethics Statement

All experimental protocols were conducted in accordance with guidelines for the humane use of laboratory animals and were approved by the animal ethics committee of the Federal University of Minas Gerais (protocol # 051/08).

### Animals

We used 10-12-week-old male Wistar rats obtained from the University animal facility. Free access was allowed to standard diet and tap water. The animals were divided in two groups named paraquat-treated and time-matched vehicle control group. The animals were dosed with a single intraperitoneal injection of paraquat (20 mg.Kg^-1^, Syngenta, São Paulo, SP, Brazil) dissolved in saline. This dose of paraquat was previously found to induce ALI in rats [16]. 24 hours after paraquat poisoning the rats showed weight loss, irregular breathing, dyspnea, pulmonary edema and increased lipid peroxidation in plasma, kidney and lung confirming the development of ALI in our animals [16]. In some experiments, the soluble tumor necrosis factor (TNF) receptor fusion protein (etanercept; 1 mg.Kg^-1^) was injected subcutaneously 1 h before paraquat, and 1h or 6 hs after paraquat. Animals were killed 24 hours after paraquat administration by decapitation. The aorta was removed and bronchoalveolar lavage (BAL), blood and lung tissue were carefully collected to be used in experiments.

### Bronchoalveolar lavage

Bronchoalveolar lavage (BAL) was performed to obtain leukocytes from alveolar spaces [17] and to measure lung edema [18]. The trachea was exposed and a 1.7-mm-outside diameter polyethylene catheter was inserted. BAL was performed by washing the lungs three times with three different 1-ml aliquots of phosphate buffered saline (PBS). BAL samples (2.0 ml each) were centrifuged at 600 x *g* for 5 minutes at 4^°^C. The supernatant was stored to analyze total protein content and the cell pellet used to evaluate the number of infiltrating leukocytes. The pellet containing cells from the BAL fluid was resuspended in 100 µl of PBS containing 3% bovine serum albumin (BSA) and an aliquot diluted in Turk solution 1:10. Total leukocyte counts were then performed in a Neubauer chamber using an optical microscope (Standard 25, Zeiss, Germany). Differential counts were obtained from cytospin (Shandon III) preparations by evaluating the percentage of neutrophils on a slide stained with Panoptic. Analysis was carried out under an immersion objective 100X and at least 300 cells were counted. Leukocyte types were defined using standard morphological criteria. Total protein content was measured by the Lowry method [19].

### Quantification of Neutrophil and Macrophage Accumulations in Lung Tissue

The extent of neutrophil accumulation in lung tissue was measured by assaying myeloperoxidase (MPO) activity, as previously described [20] and the infiltration of mononuclear cells into the lungs was quantified by measuring the levels of the lysosomal enzyme N-acetyl-β-D-glucosaminidase (NAG) which is present in high levels in activated macrophages [21]. Briefly, before lung removal, the pulmonary vasculature was perfused with 3 ml of PBS through the right ventricle, and the organ was removed and frozen at -80^°^C. After thawing, the tissue (0.1 g of tissue per 1.9 ml of buffer) was homogenized in a pH 4.7 buffer 1 (0.1 M NaCl, 0.02 M Na_2_PO_4_, 0.015 M Na _2_EDTA), centrifuged at 12.000 x *g*, 4^°^C for 10 minutes and the pellet was resuspended in 200 µl of buffer 1 + 1.5 ml of 0.2% NaCl solution + NaCl 1.6% and glucose 5% and thereafter was again homogenized. This solution was divided for MPO and NAG measurements.

### Myeloperoxidase (MPO) Activity Measurement

After further centrifugation (12.000 x *g* at 4^°^C for 15 minutes), the pellet was resuspended in 0.05 M Na _3_PO_4_ buffer (pH 5.4) containing 0.5% hexadecyl-trimethylammonium bromide (HTAB; Sigma, St. Louis, MO, USA) and re-homogenized. Samples were transferred into 1.5-ml microtubes followed by three freeze-thaw cycles using liquid nitrogen. Then they were centrifuged at 12.000 x *g*, 4^°^C for 15 minutes to perform the assay. The assay employed 25 µl of 3.4-5.6-tetramethylbenzidine (TMB; Sigma, St. Louis, MO, USA), dissolved in dimethyl sulfoxide (DMSO; Merck, Darmstadt, Germany) at a final concentration of 1.6 mM, 100 µl of H_2_O_2_, dissolved in phosphate buffer (pH 5.4) containing HTAB in a final concentration of 0.002% vol/vol and 25 µl of sample obtained. The reaction was started at 37^°^C for 5 minutes in a 96-well microplate by adding the supernatant and the TMB solution. After that, H_2_O_2_ was added and followed by a new incubation at 37^°^C for 5 minutes. The reaction was stopped by adding 100 µl of 1 M H_2_SO_4_ and quantified at 450 nm in a spectrophotometer (E_max_; Molecular Devices, Sunnyvale, CA, USA). Results were expressed as change in absorbance (optical density, OD) per milligram of wet tissue.

### N-acetyl-β-D-glucosaminidase (NAG) Activity Measurement

After a further centrifugation (12.000 x *g* at 4^°^C for 15 minutes), the pellet was resuspended in 0.9% NaCl solution, containing 0.1% Triton X-100 (Promega, Madison, WI, USA) and re-homogenized to perform the assay. Samples (100 µl) were incubated for 10 min at 37^°^C with 100 µl of substrate p-nitrophenyl-N-acetyl-β-D-glucosaminidase (Sigma, St. Louis, MO, USA) prepared in citrate/sodium phosphate buffer (0.1 M citric acid, 0.1 M Na_2_HPO_4_; pH 4.5) to yield a final concentration of 2.24 mM. The reaction was stopped by the addition of 100 µl of 0.2 M glycine buffer (0.8 M glycine, 0.8 M NaCl, 0.8 M NaOH; pH 10.6). Hydrolysis of the substrate was quantified at 405 nm in a spectrophotometer (E_max_; Molecular Devices, Sunnyvale, CA, USA). NAG activity was expressed as change in OD per milligram of wet tissue.

### Quantification of Neutrophils in blood

After sacrifice of the animals, 20 µl of blood was collected and mixed with Turk solution for total number of cells counting in a modified Neubauer chamber and 10 µl of blood was collected for evaluating the percentage of neutrophils on a slide stained with May-Grunwald-Giemsa.

### Assessment of cytokine concentrations in serum

TNF-α and interleukin 1beta (IL-1β) concentration were measured in serum of animals, using ELISA with commercially available antibodies and according to the instructions supplied by the manufacturer (R&D Systems, Minneapolis, MN, USA). Serum was obtained from coagulated blood (15 min at 37^°^C, then 30 min at 4^°^C) and stored at -20^°^C until further analysis. Serum samples were analyzed at a 1:1 dilution in the assay dilution buffer.

### Rat aortic rings preparation and mounting

The animals were killed 24 hours after paraquat administration; the thoracic aorta was carefully removed and cleaned of fat and connective tissue, as previously described [22]. Segments of 2.0-3.0 mm in length were removed and placed into Krebs–Henseleit solution of the following composition (mM): NaCl, 135; KCl, 5; KH_2_PO_4_, 1.17; NaHCO_3_, 20; MgSO_4_, 1.4; CaCl_2_, 2.5 and glucose 11. The segments were then mounted on a myograph at 37^°^C and continuously gassed with carbogenic mixture (95% O_2_ and 5% CO_2_), under a tension of 1.0 g, for 1 h equilibration period. The presence of a functional endothelium was assessed by the ability of acetylcholine (1 µM) to induce more than 80% relaxation of vessels pre-contracted with phenylephrine (0.1 µM). In certain experiments the endothelium was removed mechanically, by rubbing the intimal surface, as previously described [23]. Phenylephrine was added in increasing cumulative concentrations (0.0001-10 µM). In some experiments, after 30 min washing, the vessels were incubated for 30 min with the non-selective inhibitor of nitric oxide synthase (NOS), L-N^G^-Nitroarginine Methyl Ester (L-NAME, 300 µM, Sigma, St. Louis, MO, USA), or the selective inhibitor of endothelial nitric oxide synthase (eNOS), N^G^-Nitro-L-Arginine (L-NNA, 1 µM, Sigma, St. Louis, MO, USA) or the selective inhibitor of inducible nitric oxide synthase (iNOS), L-N^6^-(1-Iminoethyl) lysine hydrochloride (L-NIL, 10 µM, Calbiochem, San Diego, CA, USA) and a second cumulative concentration–response curve for phenylephrine was constructed and compared with the first one. Concentration–response curves were also constructed for acetylcholine (0.001-100 µM) in vessels pre-contracted with sub-maximal concentration of phenylephrine (0.1 µM). In another set of experiment, the acute *in vitro* effect of paraquat was evaluated. To this end, concentration–response curves for acetylcholine (0.001-100 µM) were constructed in vessels removed from control animals and pre-incubated with paraquat (5 µM) for 20 minutes. Mechanical activity recorded isometrically by a force transducer (World Precision Instruments, Inc., Sarasota, FL, USA) was fed to an amplifier-recorder (Model TBM-4; World Precision Instruments, Inc.) and to a personal computer equipped with an analogue-to-digital converter board (DI-720; Dataq Instruments, Inc.), using Windaq data acquisition/recording software (Dataq Instruments, Inc.).

### Nitrite measurement

Nitric oxide (NO) was determined indirectly measuring the concentration of nitrite by using 2,3-diaminonaphthalene (DAN; Sigma, St Louis, MO, USA) fluorescent method according to [24]. Briefly, endothelium-intact segments of aorta were maintained in tubes containing Krebs–Henseleit solution (1 ml) at 37^°^C for 15 minutes. After removal of the perfusate the segments of aorta were left in the presence of 1 ml Krebs–Henseleit solution containing L-NIL (10 µM) for further 15 minutes. For nitrite measurements 100 µl of perfusate in each condition was mixed with 10 µl of 0.05 mg/ml DAN. After 10 minutes incubation at 20°C protected from light, the reaction was stopped with 10 µl of 2.8 M NaOH. Formation of fluorescent product was measured using a fluorescent plate reader (Cary Eclipse Microplate reader, VARIAN, Inc.) with excitation at 360 nm and emission read at 440 nm with a gain setting at 100%.

### Western Blot Analysis

Western blot was performed as previously described [16]. Briefly, the frozen aorta with the endothelial layer was homogenized in lysis buffer (in mM): 150 NaCl, 50 Tris-HCl, 5 EDTA.2Na, and 1 MgCl_2_ containing 1% Triton X-100 and 0.5% SDS plus a cocktail of protease inhibitors (SigmaFAST, Sigma, St. Louis, MO, USA) and phosphatase inhibitors (20 mM NaF; 0.1mM Na _3_VO_4_). 40 µg of protein were denatured and separated in denaturing SDS/7.5% polyacrylamide gel. Proteins were transferred onto a polyvinylidene fluoride membrane (PVDF - Immobilon P; Millipore, Billerica, MA, USA). Blots were blocked at room temperature with 3% non-fat dry milk in PBS plus 0.1% Tween 20 (PBS-T) before incubation with rabbit polyclonal anti-iNOS; (1:2000; Santa Cruz Biotechnology, Santa Cruz, CA, USA), rabbit polyclonal anti-eNOS; (1:2000; Sigma, St Louis, MO, USA) or mouse monoclonal anti-β-actin (1:2500; Santa Cruz Biotechnology, Santa Cruz, CA, USA) at room temperature. The immunocomplexes were detected by chemiluminescent reaction (ECL^+^ kit, Amersham, Les Ulis, France) followed by densitometric analyses with software ImageJ.

### Confocal microscopy

Immunolocalization of iNOS was performed according to [25] with some modifications. Briefly, cold methanol-DMSO (1: 1 v/v) fixed cryosections (10 µm) of the thoracic aorta from control and paraquat-poisoned rats were fixed in cold acetone for 15 minutes and rinsed in PBS wash buffer (1% BSA + 0.3% Triton X-100, in PBS). Following appropriate blocking procedures (3% BSA + 0.3% Triton X-100 in PBS, 30 minutes), cross reactivity of secondary antibodies with the alternating primary antibodies was removed. Slides were incubated with rabbit anti-iNOS (Santa Cruz Biotechnology, 1:100) overnight at 4°C followed by incubation with goat anti-rabbit secondary antibody conjugated with Alexa Fluor 633 (1:200, Invitrogen, Carlsbad, CA, USA) for 1 h. The sections were examined with a Zeiss LSM 510 confocal microscope (Thornwood, NY, USA) with excitation at 633 nm and emission at 650 nm. Fluorescence intensity (measured using ImageJ® software 1.42q; Wayne Rasband, NIH) in paraquat-poisoned aorta was expressed as relative fluorescence intensity (arbitraries units). Ten fields per slide of endothelial layer were measured. The mean of fluorescence from each slide was plotted and analyzed using GraphPad Prism 4 (Graphpad Software Inc., La Jolla, CA, USA).

### Statistical analysis

Results are expressed as means ± SE. Two-way analysis of variance (ANOVA) was used to compare concentration–response curves. Student’s t-test and One-way ANOVA was used in the other experiments. All statistical analyses were considered significant when p < 0.05.

## Results

### Paraquat-induced mortality

A mortality of approximately 25% was observed, 24 hs after paraquat administration. However, no mortality was seen after etanercept given before paraquat exposure, or 1h or 6 hs after paraquat exposure.

### Lung and systemic inflammation

**Figure 1:**
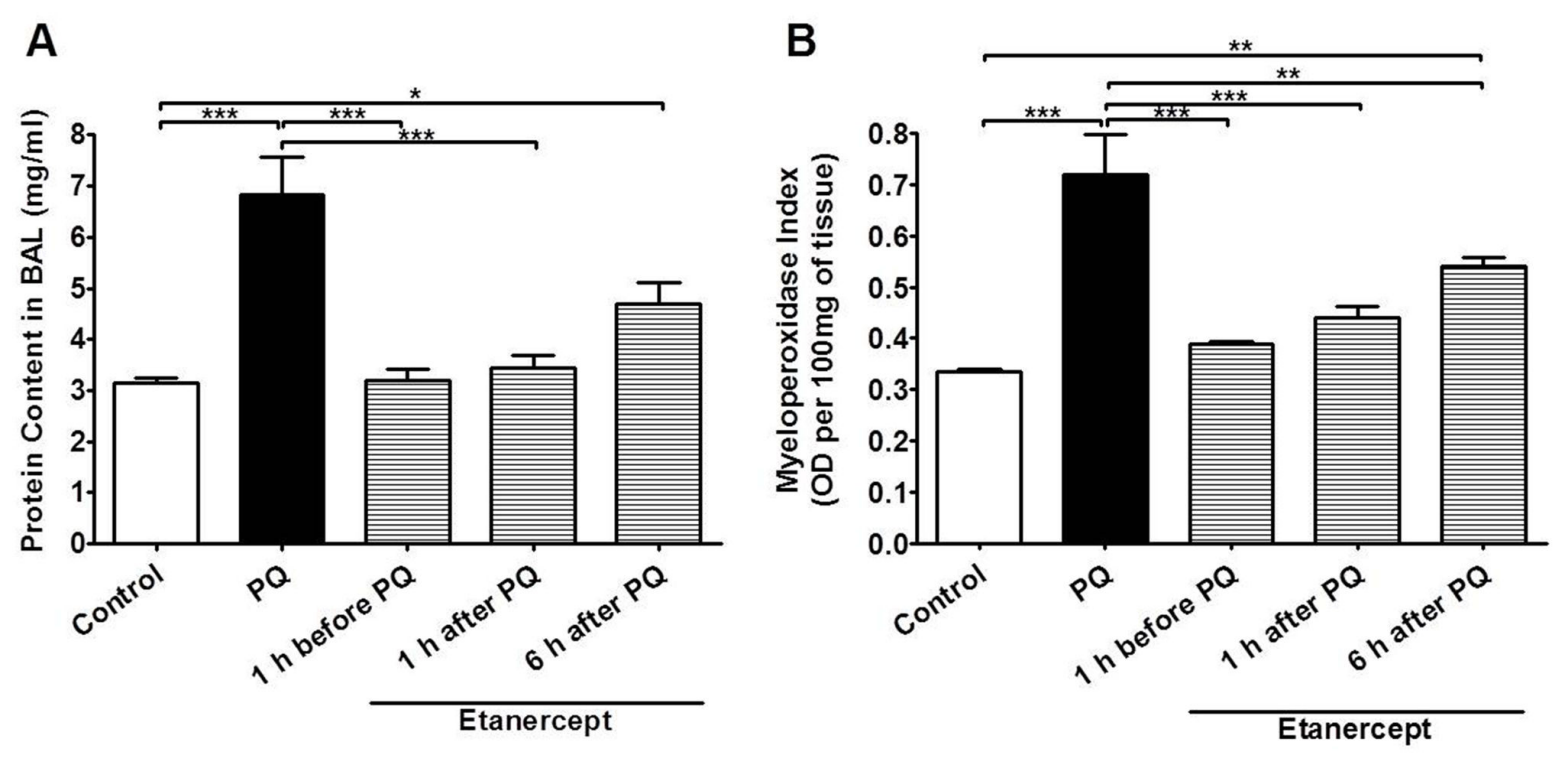
Paraquat-poisoning produces TNF-α-mediated edema and neutrophil accumulation in the lungs. Pulmonary permeability (**A**) and neutrophil accumulation in the lungs (**B**) of control, paraquat-poisoned (PQ) and paraquat-poisoned rats after treatment with etanercept. The total protein content was used as an index of protein leakage due to alveolar-microvascular membrane injury. Myeloperoxidase (MPO) activity was used as an index of neutrophil influx into the lungs. Results are shown as protein content (mg/ml) or MPO index and represent the mean ± SE of five animals in each group. *p<0.05; **p<0.01 and ***p<0.001.

**Figure 2:**
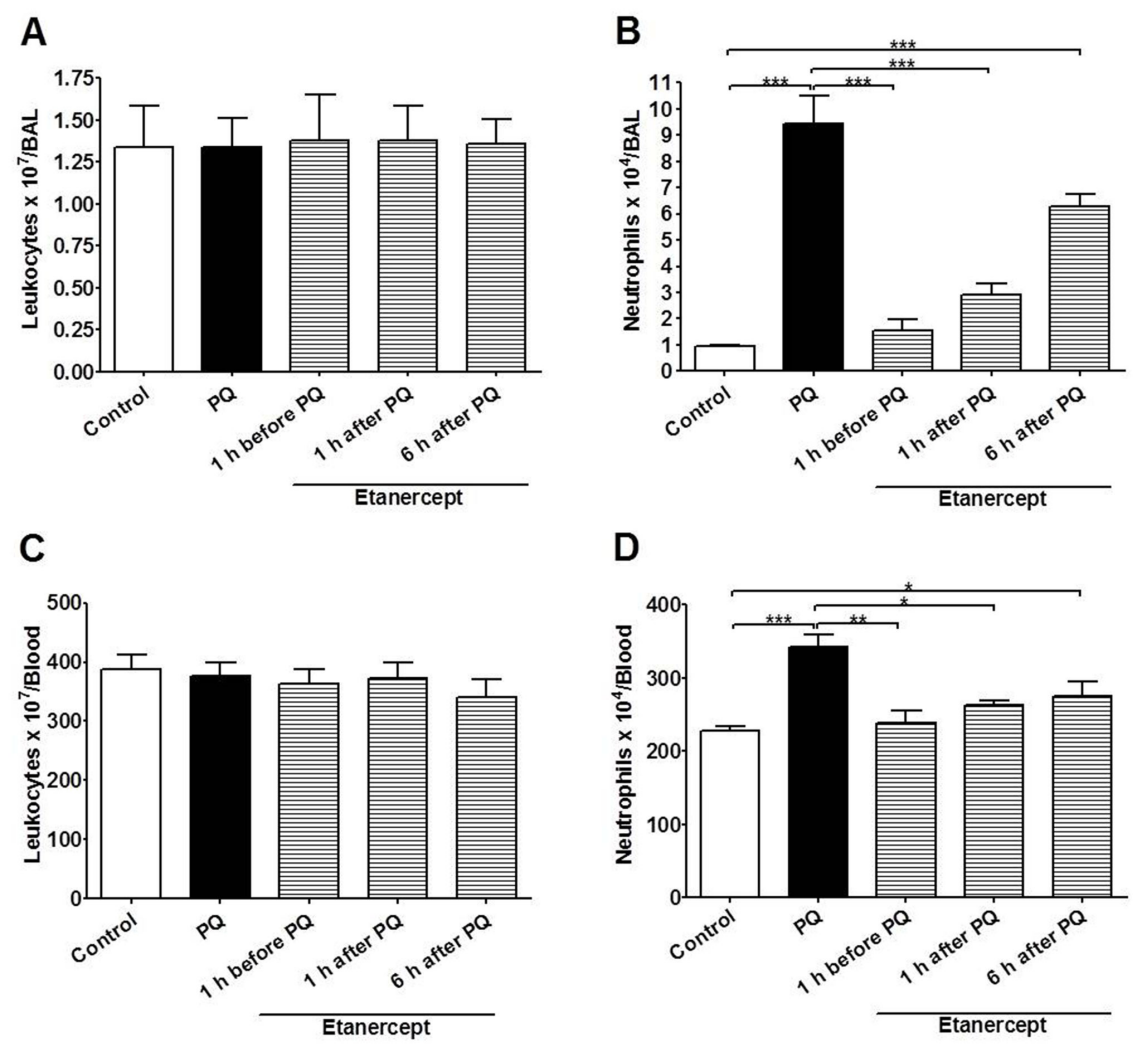
Paraquat produces accumulation of neutrophils in bronchoalveolar lavage (BAL) and neutrophilia. Effect of paraquat-poisoning (PQ) on total number of leukocytes and neutrophil in BAL (**A**, **B**) or in blood (**C**, **D**). TNF-α blockade by etanercept restore neutrophil number to the control level in BAL (**B**) and blood (**D**). Results are shown as the mean ± SE of five animals in each group. *p<0.05; **p<0.01 and ***p<0.001.

**Figure 3:**
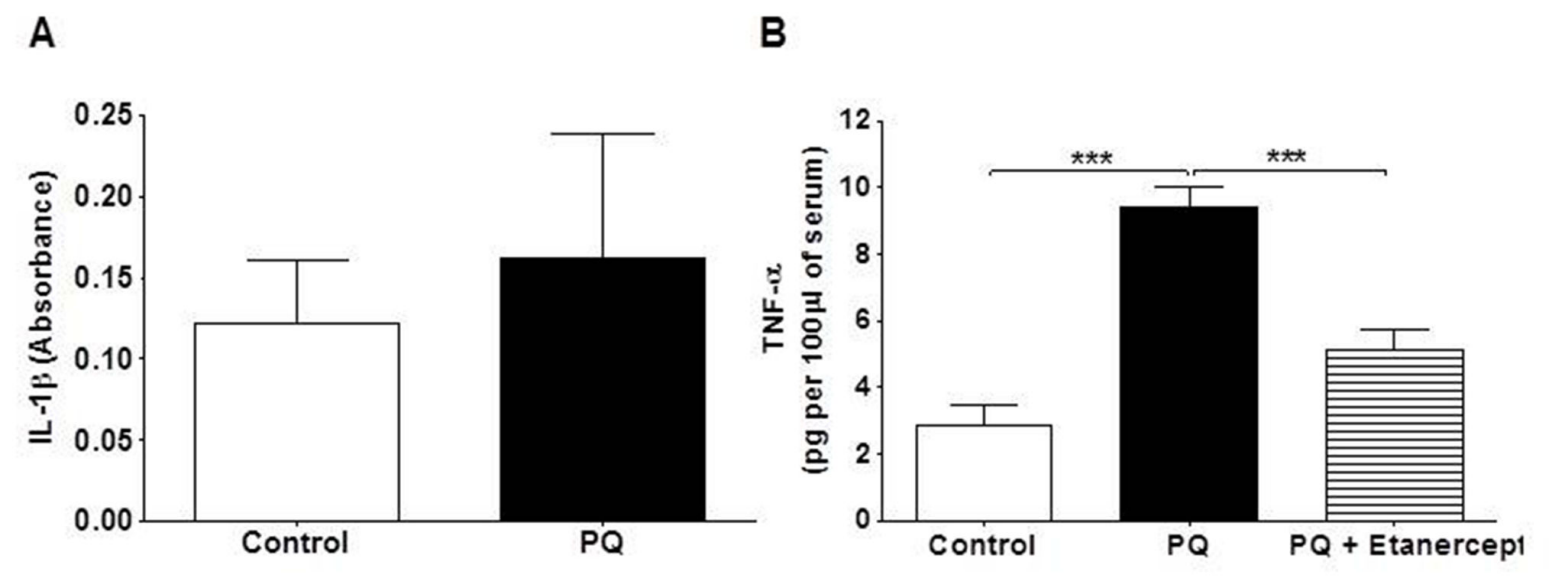
TNF-α concentration is increased in the serum of paraquat-poisoned animals. Effect of paraquat-poisoning (PQ) on (**A**) IL-1β (n=11-12) and (**B**) TNF-α (n=5) concentration in serum. Results are shown as the mean ± SE ***p<0.001.

Paraquat poisoning caused considerable lung edema (Figure 1A) and accumulation of neutrophils in lung tissue (Figure 1B) and BAL (Figure 2B). There was also significant blood neutrophilia (Figure 2D). However, there was no macrophage infiltration in the lung, as assessed by measuring NAG (OD) per 100 mg of tissue: 0.97 ± 0.07 and 0.92 ± 0.07, control and paraquat, respectively, and the total number of leukocytes in BAL and blood were unaltered (Figure 2A and Figure 2C). The concentrations of the cytokines IL-1β and TNF-α were evaluated in serum of control and paraquat-poisoned rats. Paraquat poisoning produced a significant increase in the concentration of TNF-α (Figure 3B) in the serum but had no effects on the concentration of IL-1β at 24 h (Figure 3A). Treatment of animals with etanercept reduced pulmonary permeability, neutrophil accumulation in lungs, BAL and blood (Figure 1A, B and Figure 2B,D) and TNF-α in serum (Figure 3B) to the control level.

### Vascular Response

**Figure 4:**
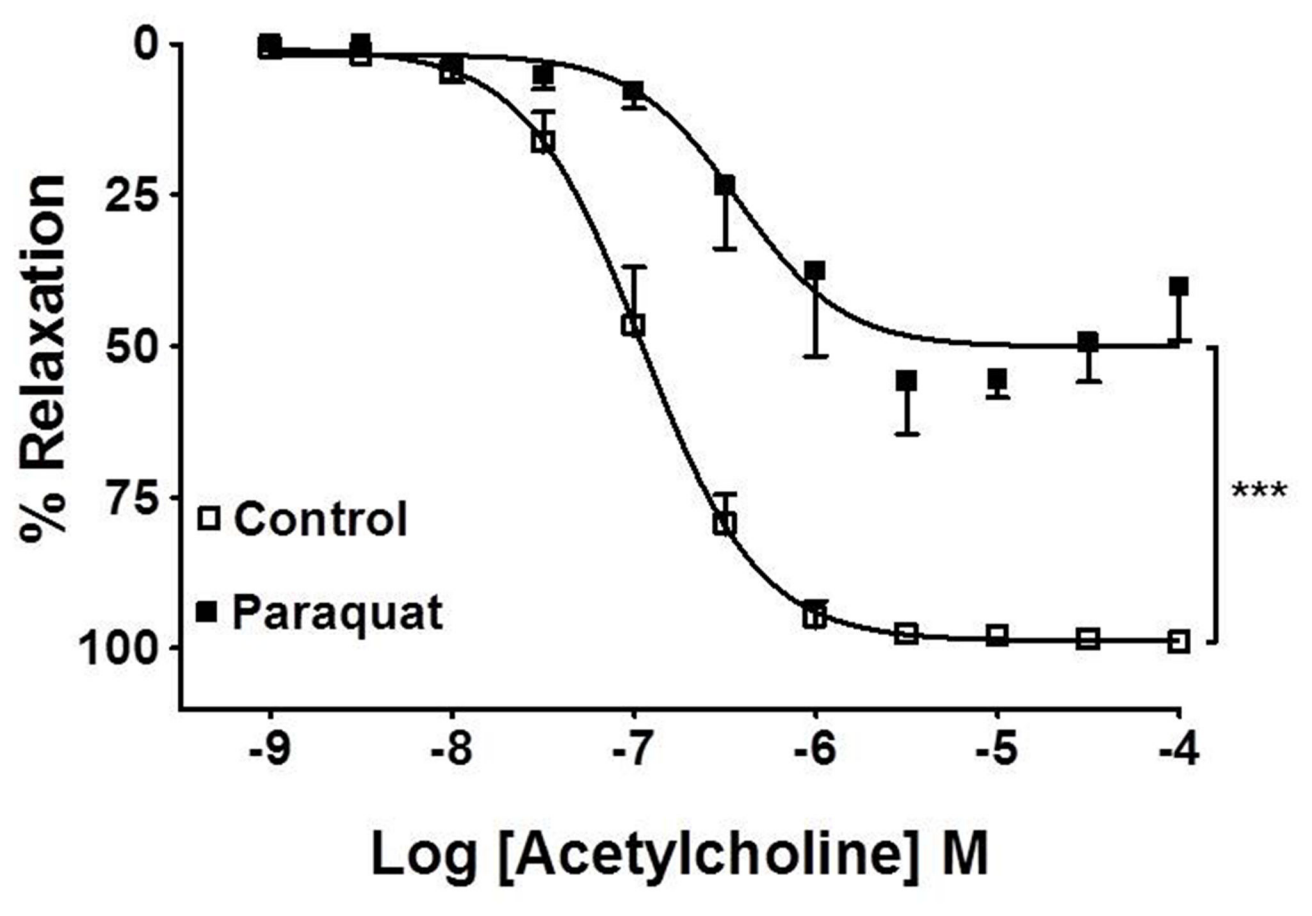
*In vitro* effect of paraquat in aortic rings. Effects of *in vitro* treatment of endothelium-intact aortic rings with paraquat (5 µM) for 20 min on vasodilation induced by acetylcholine. The values are mean ± SE from five experiments. ***p<0.001.

Acetylcholine induces endothelial-dependent vasorelaxation *in vitro*. The vasorelaxant response of the rat aorta to acetylcholine was impaired in isolated vessels treated *in vitro* with paraquat (5 µM) for 20 minutes (Figure 4). Therefore, paraquat causes endothelial dysfunction *in vitro*.

**Figure 5:**
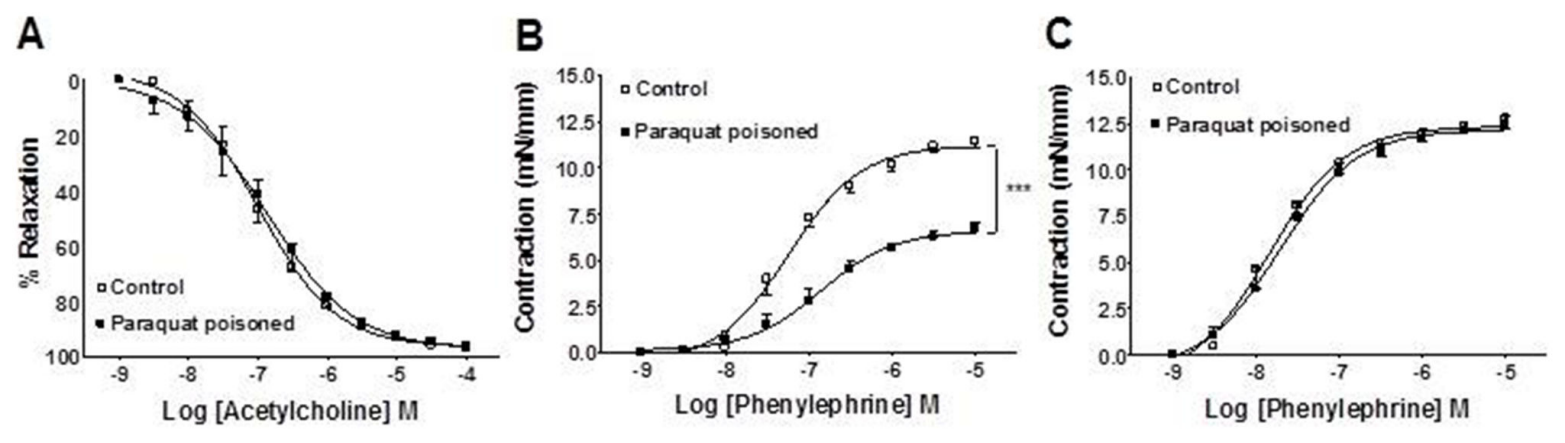
*Ex vivo* vascular effects of paraquat-poisoning. Vasodilator effect of acetylcholine in endothelium-intact aortic rings from control and paraquat-poisoned animals (**A**). Contractile response to phenylephrine in endothelium-intact (**B**) and endothelium-denuded (**C**) aortic rings from control and paraquat-poisoned animals. The values are mean ± SE from five experiments. ***p<0.001.

In order to access systemic vascular responsiveness 24 hs after paraquat poisoning, aortas were stimulated with relaxant and contractile agents. As shown in Figure 5A, acetylcholine-induced vasorelaxation was not different in control animals and those subjected to ALI by paraquat. In contrast, phenylephrine-induced vasoconstriction was severally impaired in endothelium-intact aortic rings from the ALI group (Figure 5B). Removal of the endothelium restored vascular response of aortas from the ALI group to the level of control vessels (Figure 5C), suggesting a role of endothelial factors in the impaired phenylephrine-induced vasoconstriction.

**Figure 6:**
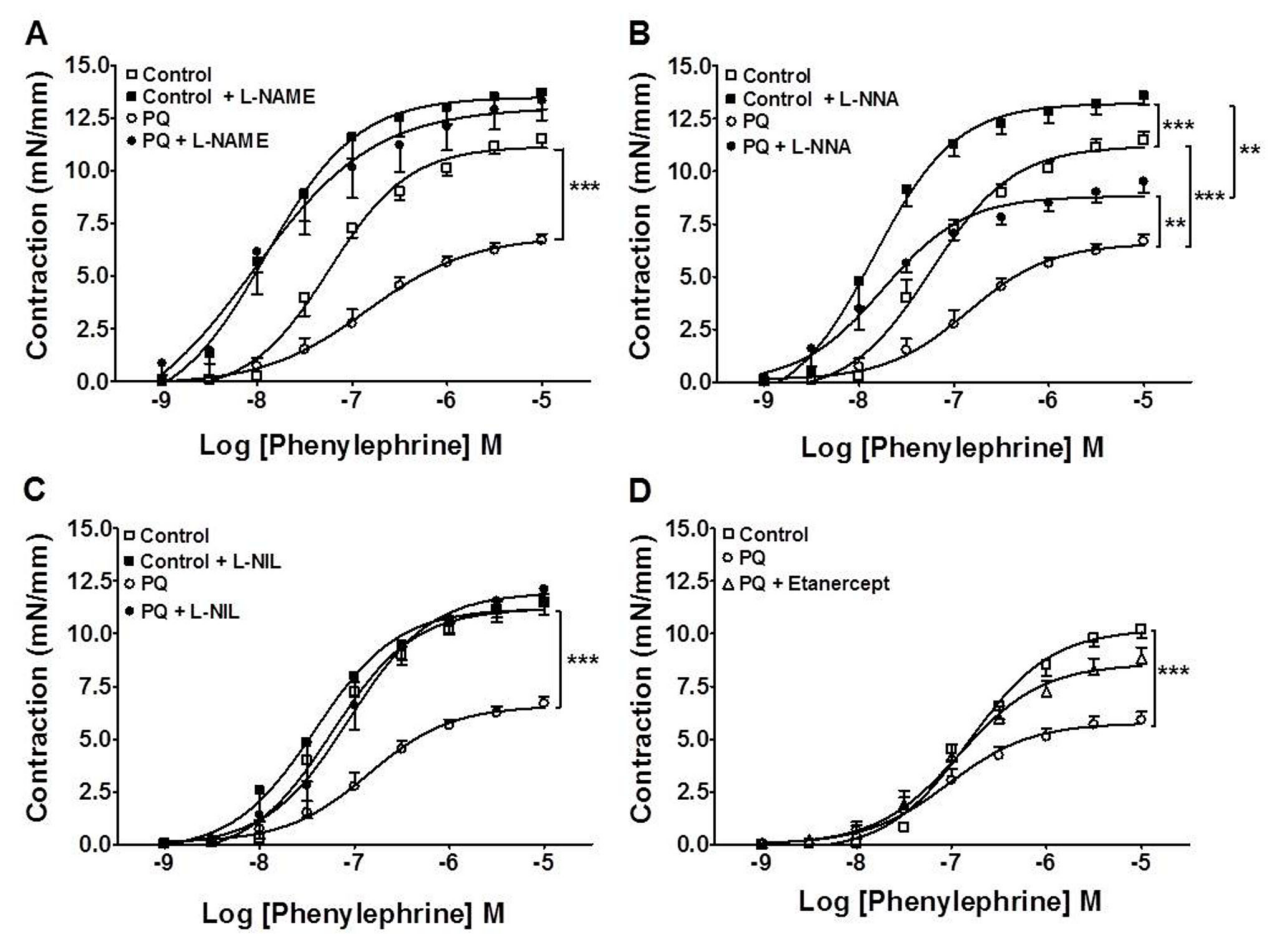
Nitric oxide and TNF-α mediate vascular dysfunction in paraquat-poisoned animals. Effect of (**A**) L-NAME (300 µM), (**B**) L-NNA (1 µM), (**C**) L-NIL (10 µM) and (**D**) *in vivo* treatment with etanercept on phenylephrine-induced contraction in endothelium-intact aortic rings from control and paraquat-poisoned (PQ) animals. The values are mean ± SE from five experiments. **p<0.01; ***p<0.001.

**Figure 7:**
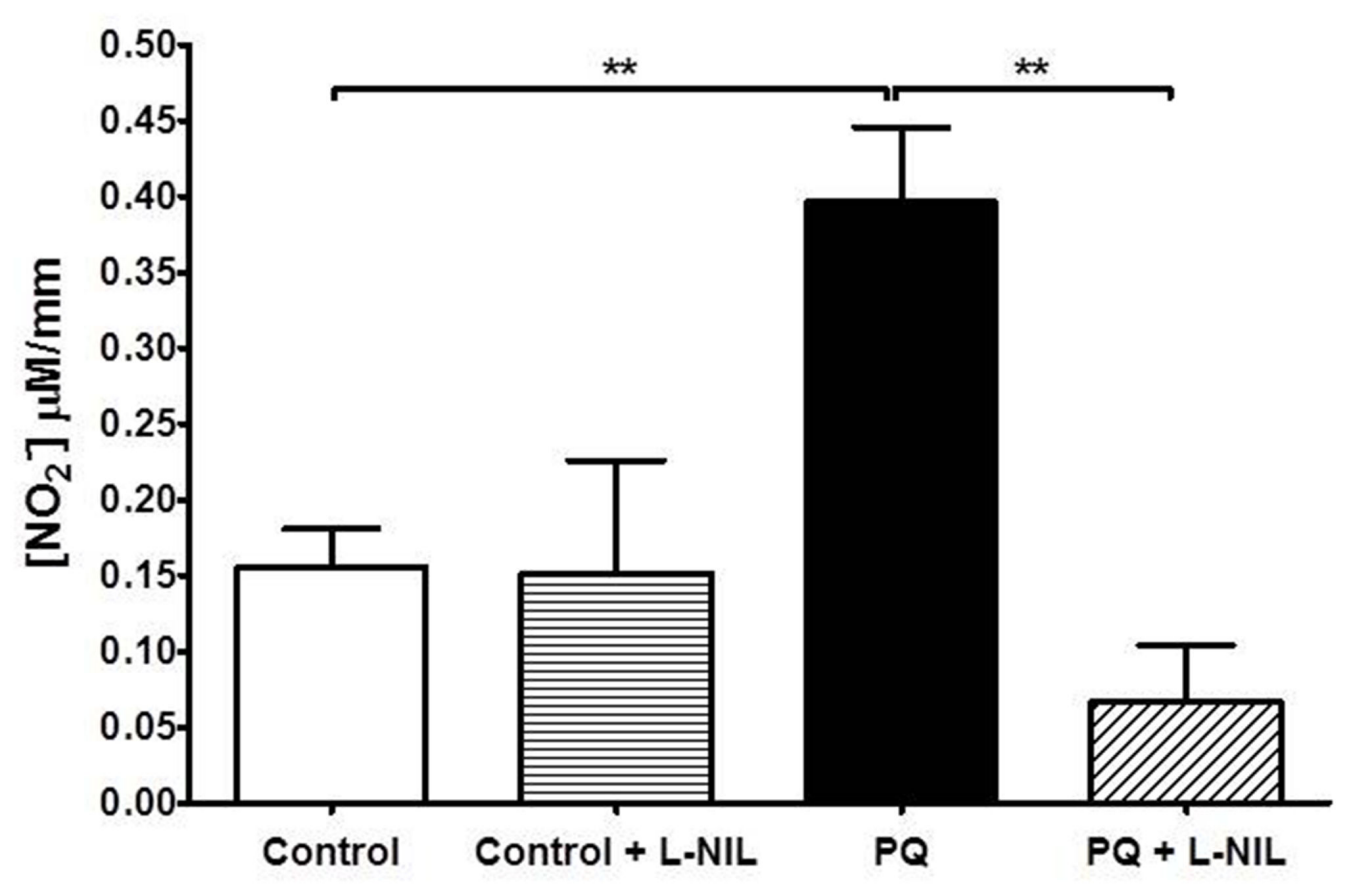
Paraquat-poisoning increases basal production of nitric oxide (NO) in the aorta. Basal production of NO in endothelium-intact aortic rings removed from control and paraquat-poisoned (PQ) animals in the presence or in the absence of L-NIL (10 µM). Results are shown as the mean ± SE from at least five experiments. **p<0.01.

Non-selective inhibition of NOS with L-NAME (300 µM) also leveled vascular contractions of the control and ALI groups (Figure 6A). Selective inhibition of eNOS with L-NNA (1 µM) increased the contractile response in both control and ALI groups. However, contraction of vessels from ALI rats remained impaired (Figure 6B). Interestingly, selective inhibition of iNOS *in vitro* with 10 µM L-NIL (Figure 6C) or *in vivo* inhibition of TNF-α by etanercept (Figure 6D) completely restored contractile response to phenylephrine in endothelium-intact aortic rings from ALI animals, as seen with endothelial removal and L-NAME. However, L-NIL did not alter contractile response in endothelium-denuded aortic rings from both groups (data not show). In separate experiments, we evaluated basal production of nitrite as an indicator of NO production. As seen in Figure 7, there was higher basal NO production in endothelium-intact aorta from paraquat-poisoned rats, as compared to control animals. Pre-incubation of the vessels with L-NIL (10 µM) restored basal values of NO production in paraquat-treated group to the same level of the control. Therefore, together the above results show an impaired contractile response probably due to an increase in iNOS expression and NO production in endothelial cells. Moreover, these results show that *in vivo* activation of TNF receptors are necessary for contractile dysfunction.

### eNOS and iNOS expression

**Figure 8:**
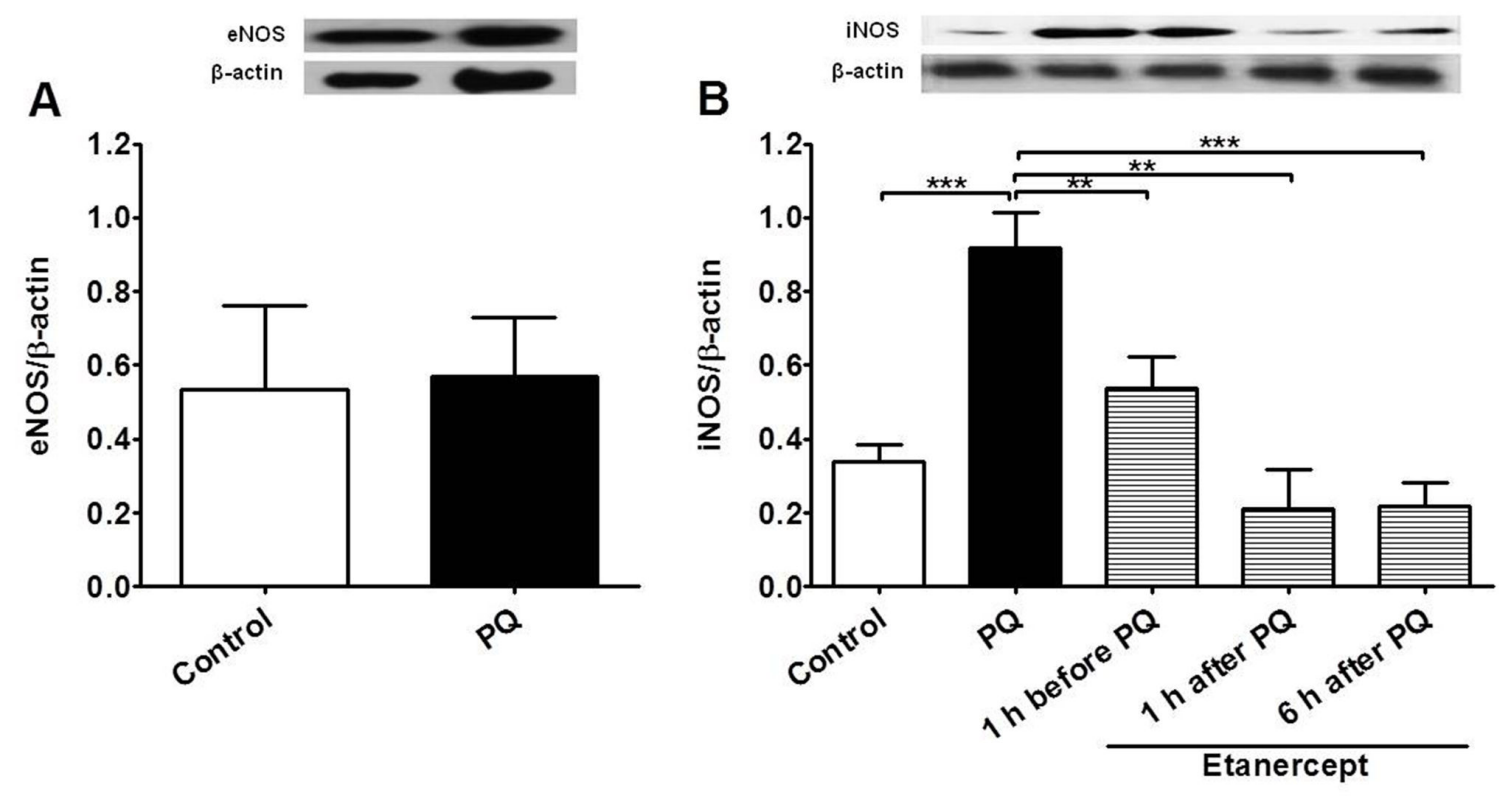
TNF-α-dependent increases of iNOS expression in aortas from paraquat-poisoned animals. Western blot analysis of (**A**) eNOS in endothelium-intact aortic rings from control and paraquat-poisoned (PQ) rats and (**B**) iNOS in endothelium-intact aortic rings from control, paraquat-poisoned and paraquat-poisoned animals after *in vivo* treatment with etanercept. Bar graphs represent mean ± SE of five experiments. Images are representative blots from four separate experiments. **p<0.01 and ***p<0.001.

**Figure 9:**
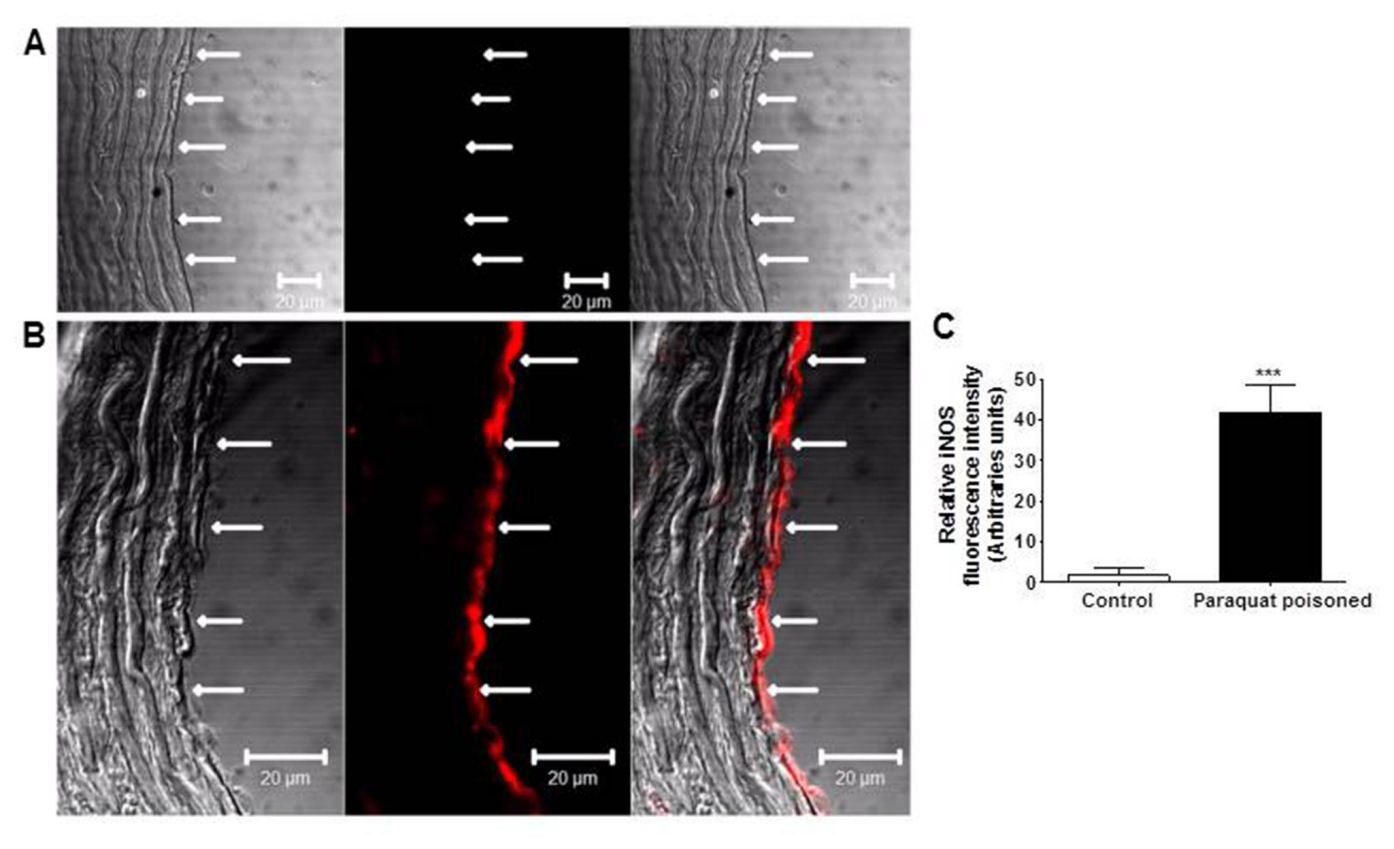
Paraquat-poisoning increases expression of iNOS in the vascular endothelial cell layer. Imunofluorescence detection of iNOS (**A**) in endothelium-intact aortic rings from control and (**B**) paraquat-poisoned animals. Immunostaining for iNOS is shown only in endothelial cells (arrows) of vessels from paraquat-poisoned animals. (**C**) Graphical representation of the relative iNOS fluorescence intensity in endothelial cells. Images are representative of five animals for each group. ***p<0.001.

Next we investigated whether there was enhanced expression of NOS in endothelial cells of aortas 24 hs after paraquat poisoning and whether TNF-α was relevant for this expression. Expression of eNOS and iNOS was evaluated by Western blot. As shown in Figure 8, the expression of eNOS was similar in endothelium-intact aortic rings from control and paraquat-poisoned animals. In contrast, the level of iNOS expression in aortic rings from paraquat-poisoned animals was approximately 4.0-fold higher compared to control vessels. Confocal analysis to immunolocalize iNOS concurred with our previous findings and showed intense staining for this isoform only in the endothelial cells layer of the aortas from paraquat-poisoned animals. No immunostaining for iNOS was observed in control group (Figure 9). Treatment of animals with etanercept reduced iNOS-dependent vascular dysfunction and iNOS expression to levels similar to those found in control animals, as shown by functional experiments (Figure 6D) and Western blot analysis (Figure 8B).

## Discussion

The major ﬁndings of our study can be summarized as follows: 1) Paraquat induced significant pulmonary and systemic inflammation when given to rats; 2) Paraquat administration caused marked contractile, but not relaxant, dysfunction of the aorta; 3) Vascular dysfunction was associated with increased iNOS expression, enhanced NO production and was blocked by selective iNOS inhibitors; 4) TNF-α is necessary for iNOS expression and iNOS-dependent vascular dysfunction. Therefore, our studies suggest a central role of TNF-α in driving paraquat-associated vascular contractile dysfunction.

Paraquat is one of the most clinically significant herbicides in terms of morbidity and mortality. Moreover, most treatments used for paraquat poisoning are not effective, suggesting a major need for novel therapies [26]. The pathogenesis of paraquat toxicity consists of two distinct phases. The initial stage involves acute damage to several organs and death may occur during this period and is associated with pulmonary, renal, and circulatory failure. Patients surviving this stage may evolve to the second stage, which is characterized by damage almost exclusively to the lungs. Extensive pulmonary fibrosis ensues, resulting in dyspnea, cyanosis, and eventually death from respiratory failure (for review see 1).

Although circulatory failure is present and contributes to increased mortality in the early stage of paraquat poisoning, there are little data available on vascular responsiveness after toxic exposure to paraquat. In this study, we showed that paraquat induces pulmonary and systemic inflammation, characterized by neutrophilia, pulmonary neutrophil influx and elevated circulating levels of TNF-α. It is largely accepted that paraquat accumulates preferentially in lungs due to an active polyamine uptake transport systems that concentrate paraquat rapidly into the type II epithelial cells of the alveoli [2]. In lungs paraquat induces oxidative stress [27,28] and inflammation [29,30,31,32]. Indeed, the pathogenesis of ALI induced by paraquat is driven by an aggressive inflammatory reaction with increased polymorphonuclear cells [32] and TNF-α [33] that increases the permeability of the alveoli-capillary unit. Our results are in accordance with the statements above as we found increased lipid peroxidation in lungs (data not shown), accumulation of neutrophils in lungs and BAL and increased pulmonary permeability. The increased number of polymorphonuclear cells and the lung edema observed in this study are consistent with other studies in which ALI is induced by distinct stimuli [11,34,35].

Although pulmonary inflammation has extensively been investigated in paraquat-induced ALI, results regarding systemic inflammation have not been reported yet. In the present study, we showed that, in addition to local inflammation, neutrophil number and TNF-α levels were increased in the systemic circulation, which is consistent with the occurrence of systemic inflammation. It is interesting to note that pulmonary concentration of paraquat can be 6 to 10 times higher than those in the plasma, and that the compound is retained in the lungs even when blood levels start to decrease [3]. Therefore, although systemic exposure to paraquat may contribute to systemic inflammation, it is more likely that systemic inflammation is a consequence of the excessive pulmonary response to high levels of the herbicide.

There are several studies which have shown that paraquat can cause endothelial dysfunction *in vitro*. However, there are no reported studies on the mechanisms that underlie the systemic vascular failure that develops *in vivo* after paraquat exposure. Consistent with data from the literature [12], in this work we found a strong decrease in vascular relaxant response when the aortas of animals were directly exposed to paraquat. Formation of reactive oxygen species has been implicated in the mechanistic events of *in vitro* paraquat-induced endothelial dysfunction [36,37]. However, when vessels were removed from paraquat-poisoned animals, no difference in relaxant response was found. This is substantially different from the *in vitro* findings and suggests that a direct effect of paraquat *in vivo* on endothelial cells is unlikely. In contrast to the normal relaxant response, we found a major decrease in contractile responses of the aorta to phenylephrine. Based on our results, the mechanism that underlies the decrease in responsiveness of the rat aorta to contractile agents is suggested to be the increased NO basal production as consequence of increased expression of iNOS in endothelial cells. The following results support the above idea: 1) Endothelium removal and selective inhibition of iNOS with L-NIL restored contraction from paraquat-poisoned aortas to the same level as those of control animals; 2) The basal level of NO production was increased in vessels of animals exposed to paraquat and L-NIL restored concentrations of NO to the level of control; 3) iNOS was highly expressed in the endothelial layer of the aortas from animals poisoned with paraquat but was absent in control vessels.

The cytokine TNF-α has been shown to play a major role in driving the expression of iNOS in inflammatory states [38,39]. In our experiments, there was increased systemic production of TNF-α, which is consistent with a possible role of this cytokine. It has been reported that neutrophils exposed to paraquat showed high production of TNF-α and enhanced degradation of IκB-α, allowing increased activity of NF-κB [40]. It is largely accepted that NF-κB activates transcription of various inflammatory genes, including iNOS [41,42], which produces massive amounts of NO independent of elevations in intracellular calcium level [38]. Not only was TNF-α increased after paraquat exposure, but more importantly treatment of animals with etanercept prevented lung edema, neutrophil accumulation in BAL, lungs and blood, TNF-α level in serum and iNOS expression in aorta. Notably, vascular responsiveness was recovered after blockade of TNF-α with etanercept. Together, these data support our assumption that exposure of animals to toxic doses of paraquat induces pulmonary and systemic inflammation that leads to increase in serum TNF-α and consequent TNF-α-dependent iNOS expression in the aortic endothelium, production of high amounts of NO and decreased vascular responsiveness to contractile agents.

In conclusion, paraquat poisoning produces a systemic inflammatory response with elevated levels of TNF-α. This cytokine increases the expression of iNOS in the endothelial cell layer of the aorta, increasing basal production of NO, impairing the contractile vascular response. Hence, the results found in this work are in line with the systemic vascular failure that develops *in vivo* in paraquat-poisoned patients. Moreover, our results suggest that inhibition of TNF-α by etanercept may be useful in cases of paraquat poisoning, a possibility that deserves careful clinical trials.
